# Importation of Horses

**Published:** 1892-08

**Authors:** 


### IMPORTATION OF HORSES.

The Veterinarian is frequently called upon in regard to the importation of horses into the United States, or is employed to take charge of animals imported. We should be cognizant of the law regarding it, and we think that the last Treasury decision on the McKinley law will be of interest to our readers.

### NEW TREASURY REGULATIONS.

(*Extract from United States Treasury Department's Circular No. 66, May 2, 1892.*)

It having been ascertained that animals which are crossbred and others with unknown pedigrees, have been recorded in certain registers, with the sole object of making them eligible for free entry into the United States, and as paragraph 482 of the Act of October 1, 1890, provides that no animal shall be admitted free unless pure bred of a recognized breed, the object of the law being, in the opinion of this Department and the Department of Agriculture, to exclude from free entry animals not absolutely and strictly pure bred, it is hereby directed that on and after June 1, 1892:

"No animal which is brought into the United States from foreign countries for breeding purposes, shall be admitted free of duty unless the importer furnishes a certificate of the record and pedigree *in the form hereafter given*, showing that the animal is pure bred and admitted to *full registry* in a book of record established for that breed; *that both its sire and dam were likewise recorded* in a book of record established for the same breed; and that there have been *four successive top crosses by recorded sires of that breed on the side of the dam*, together with the affidavit of the owner agent or importer that such animal is the identical animal described in said certificate of record and pedigree."

"Unless the certificate of record and pedigree herein provided for is produced, the animal shall be considered dutiable as not being pure bred of a recognized breed and duly registered in the book of record established for that breed."

"In case such certificates are not at hand at the time of the arrival of the animals, and other evidence is produced, satisfactory to the collector, showing that the animals are entitled to free entry, the collector may so admit them, taking a bond in double value of the animals for the production of the proper certificate."

The following forms have been prepared by Mr. Godfrey, Secretary of the American Hackney Horse Society, but are applicable for the entry of any registered horse, by filling in the proper name of the class of horse and the Stud Book.

This form is intended for use in passing through the Customs horses registered in Stud Book and imported into the United States from Canada and other countries. The person selling the horse mentioned hereon should sign the Vendor's Certificate and forward this Form to the Secretary, who will certify to extended pedigree.

VENDOR'S CERTIFICATE.

*This is to certify* that the animal hereinafter described has been sold by me to Mr.........................of.........................for exportation to the United States.

American No............Name ...... ................Sex..... ...(Stallion or Mare.)

English No...........(If known.) Foaled in 18.....Color..................

(Signature of Vendor).... ... ...............

Date....................189 . Address............... .......

### CERTIFICATE OF RECORD AND PEDIGREE.

(*This Certificate must bear the Official Seal of the Society.*)

| | | | | |
|---|---|---|---|---|
| PEDIGREE OF | Sire.... ... | | | |
| Name...... | No......... | Sire.......... | | |
| No......... | | No......... .. | | |
| 1st. | Dam....... | | Sire......... | |
| | No..... 2nd | Dam.......... | No.......... | Sire.......... |
| | | 3rd. | Dam.......... | No........ |
| | | | 4th. | Dam ........ |

I hereby certify that the above is a correct pedigree of............. ......
No..........., and that this animal is pure bred and has been duly registered in the book of record established by this Association for the breed of horses.

Dated at Office of the. ..........Horse Society.

.........................189 .

[Signed).......................

Secretary of the.............. .......................

### IMPORTER'S CERTIFICATE.

(*This Certificate must be signed by the importer of the animal and must contain the full name and address of the person from whom it was purchased.*)

I hereby declare that the animal purchased by me from Mr.................
of... ....................., and which I now request shall be admitted free of duty is the identical animal described in the foregoing certificate of record and pedigree.

Dated at..................Signature of Importer Owner or Agent............

.........................189 . Address........... ...............

Purchasers of horses in Canada or in Europe should be provided with similar blanks, and have them properly filled and certified by the United States Consul, in order to prevent delay and the annoyance of bonding importations upon their arrival in American ports.

### CANINE HOSPITAL.

Among the many important and interesting buildings recently started in Philadelphia, there is one of unusual interest from the particular purpose to which it is devoted. Some time since the Trustees of the University of Pennsylvania authorized the building of an hospital for dogs, the architects appointed being Frank Miles Day & Brother.

Considerable time was taken for giving the plans and the entire project the fullest consideration in order to make the building the most complete and efficient of its kind in the world, which it will be, though the first in this country. There are several in Europe, a notable one being at Berlin, which, in some particulars, this will resemble, though it will be larger and more complete.

Under the supervision of the architects it is now being erected in the grounds of the University of Pennsylvania, in West Philadelphia, and will be completed in September.

The exterior will be very attractive of Roman hydraulic pressed face-brick and pebble-dashing, with Lake Superior red-stone trimmings, the roof being of unfading green slate. It will cover an area of about 65x50 feet.

There will be two stories and a basement, the latter to contain besides the room for ordinary cellar purposes, a kitchen, with every facility for cooking the food for the dogs, heating the water for the baths, etc.

On the first floor the commodious clinic room will open into the largest apartment, that for non-contagious cases. At either side of the latter will be the mange ward and the distemper ward, both being separated from it by heavy brick partitions, the communications with all other parts of the building from these wards being effected only by going outside and around.

In the three principal wards, as also in the bath rooms connected with them, there will be polished granolithic pavements, and the walls to a height of five feet with six-inch square white enameled tiles. Above the tiling the walls and the ceiling will be of adamant plaster. The dogs will be in separate cages upon wheels, and may thus be removed for disinfection to the baths or to the clinic. From fifty to sixty of these cages will probably be in use.

There will be special bath rooms connected with all the wards, which, with all the plumbing, have been matters of great consideration, and are the best of the kind for the purposes. The bath-room for the non-contagious and surgical ward will be entirely apart from that of the mange ward. In the latter there will be two baths, one for the ordinary bathing, and a smaller one for the medicated baths. There will be nine sinks, and the drainage will, of necessity, be most carefully attended to.

Perfect systems of heating and electric lighting will be introduced, the special system of ventilation having been devised in consultation with the Department of Hygiene of the University. There will be special ventilating flues for each room, and each flue will be provided with a small electric fan, thus insuring perfect control of each separately, and perfect ventilation for all.

The second story will be of the same dimensions as the ground floor, and divided into four rooms of sizes corresponding with the wards below, to be used as biological, pathological, physiological, and historical laboratories.

Beside the specialists connected with this new branch of the University, there will be the necessary and proper staff to act as superintendent, nurses and caretakers.

It has been decided that arrangements will probably be made, whereby dogs not requiring special treatment, but belonging to persons who leave town in summer, or any other time, will be properly cared for at such times in this institution.

